# Self-made spoon-shaped tool for removal of magnetic foreign bodies from the bladder: a case report

**DOI:** 10.3389/fsurg.2025.1528819

**Published:** 2025-03-28

**Authors:** Zhi Yuan Zhang, Huan Deng, Qi Chun Liang, Yi He Wang

**Affiliations:** ^1^ Department of Urology, Shenzhen Hengsheng Hospital, Shenzhen, China; ^2^ Department of Urology, Shenzhen Bao’an Shiyan People’s Hospital, Shenzhen, China; ^3^ Division of Surgical Sciences, Bao'an Clinical Institute of Shantou University Medical College, Shenzhen, China; ^4^ The School of Graduate Studies, Lingnan University, Hong Kong, Hong Kong SAR, China

**Keywords:** magnetic beads, foreign body, minimally invasive technique, cystoscope, surgery

## Abstract

**Background:**

Foreign bodies retained in the urethra or bladder present a rare but complex challenge in adult urological practice. Magnetic beads, in particular, are difficult to manage due to their mutual attraction and the large quantities often involved. The presence of such beads complicates removal procedures, especially in male patients with a long urethra. We describe a novel and simple method for retrieving magnetic beads from the bladder.

**Case description:**

A 23-year-old man presented with painful urination after inserting approximately 40 small magnetic beads into his urethra for sexual stimulation. Pelvic computed tomography confirmed the presence of multiple metallic bodies in the bladder. Given his preference for a minimally invasive approach and opposition to open surgery, we devised a novel retrieval method. To remove the foreign bodies in a minimally invasive manner, we used orthopedic wire to create a spoon-shaped extractor, which was inserted through a resectoscopic sheath. Using direct cystoscopic visualization, the extractor successfully removed up to six beads at a time. A total of 48 beads were retrieved from the bladder, and the patient was discharged on the second postoperative day, with no complications or residual symptoms.

**Conclusions:**

The self-made extractor reduced the risks associated with removing spherical foreign bodies and shortened the overall surgical time. This new device offers valuable insights into the efficient removal of spherical objects from the bladder, making it suitable for primary care settings where conventional instruments may be limited.

## Introduction

Foreign bodies retained in the urethra or bladder present a rare but challenging issue in adult urological practice. A wide variety of foreign objects have been documented in the lower urinary tract, with a significant number being self-inserted by patients (1, 2). In adult patients, the most common reason for self-insertion of urethral foreign bodies is the pursuit of sexual gratification. Since both the urethra and glans penis share innervation from the dorsal nerve of the penis, patients can experience significant sexual gratification during urethral insertion of foreign objects (3). Among these foreign bodies, magnetic beads are particularly difficult to manage due to their large quantity and the strong magnetic attraction of magnets. This issue is further complicated in male patients, as the longer male urethra makes the surgical procedure more challenging. The successful removal of magnetic beads requires both surgical expertise and specialized instruments. In their study, Mahadevappa et al. (4) reported a total of 10 cases of intravesical foreign bodies, among which 3 cases were managed by open surgical procedures. Urologists have used a variety of tools, such as forceps, pneumovesicoscopy, and laparoscopy, to safely extract different types of foreign bodies (5, 6).

Given that our hospital is a primary care center, it is equipped solely with conventional medical instruments. Herein, we describe an innovative method devised by us for the efficient and convenient removal of such magnetic beads. In this report, we present an unusual case involving the self-insertion of a large number of magnetic beads into the urethra, which had settled in the bladder.

## Case presentation

**Figure 1:**
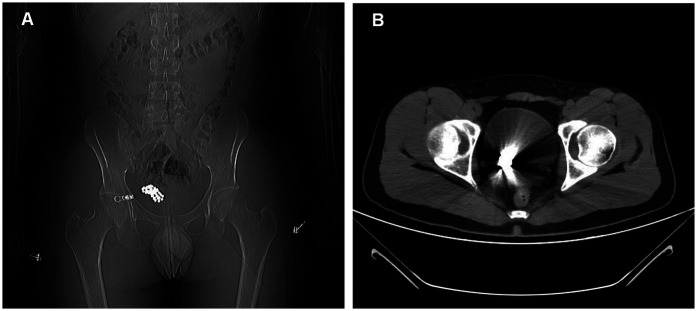
Pelvic plain computed tomography (CT) image. **(A)** Pelvic plain CT localizer radiograph showing numerous metal beads. **(B)** Plain pelvic CT scan being affected by metal artifacts.

A healthy 23-year-old man was admitted to our hospital with a history of inserting magnetic balls into his urethra 1 day ago. He reported inserting approximately 40 magnetic beads into his urethra, each measuring 5 mm in diameter. The patient did not exhibit symptoms of frequent or urgent urination, nor did he experience gross hematuria; however, he did complain of painful urination. A thorough physical examination revealed no abnormalities, and no foreign objects were palpable in the urethra. Routine urinalysis showed a white blood cell count of 47 /µl, while blood tests, liver function, renal function, and coagulation function were all within normal limits. Pelvic computed tomography (CT) confirmed the presence of multiple metallic bodies in the pelvic region (Figure 1). Given his desire to return to work, the patient expressed a strong preference for a minimally invasive approach and declined open bladder surgery.

**Figure 2:**
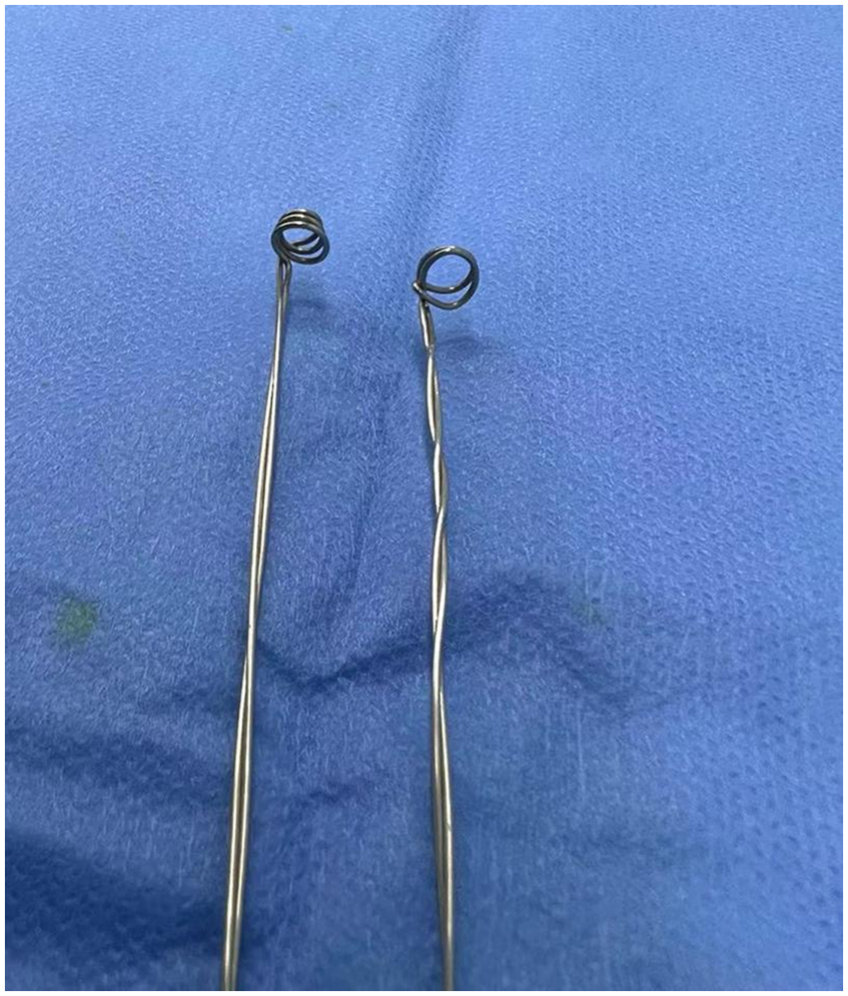
Two spoon-shaped extractors fabricated for bead removal.

Under general anesthesia, a 26 Fr sheath was carefully inserted into the urethra, revealing a cluster of metallic beads within the bladder. The primary challenge in retrieving these beads arose from their strong magnetic properties. Initially, we attempted to remove them using a resection electrode, but this proved ineffective due to the beads’ strong mutual attraction, which also caused significant deformation of the electrode. To overcome this, we devised a spoon-shaped extractor using an orthopedic wire made of non-magnetic stainless steel (Figure 2), with a diameter of 0.6 mm, which was not affected by the magnetic force of the beads.

**Figure 3:**
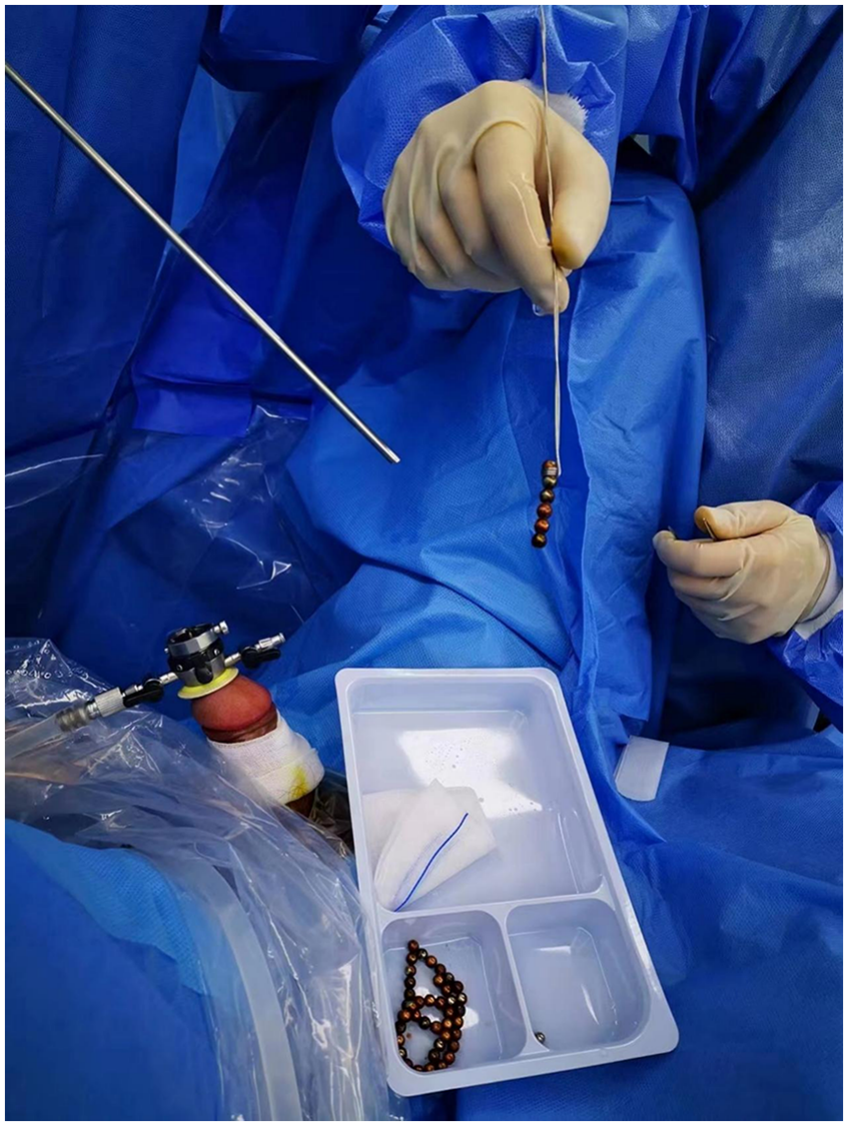
Demonstration of the tool in action, extracting several magnetic beads.

After sterilization, the extractor was inserted into the resectoscope sheath and guided under direct visualization with a cystoscope. Initially, we were able to retrieve one to three beads at a time. As the number of beads in the bladder decreased, we successfully removed up to six beads at once (Figure 3). The entire procedure lasted approximately 50 min. Following surgery, a 16 Fr urinary catheter was retained for 24 h. The patient was discharged on postoperative day 2, without painful urination or symptoms of frequent urination, urgency, or gross hematuria.

## Discussion

Foreign bodies within the urinary tract, including the bladder and the urethra can occur in both pediatric and adult populations. In this case, our patient sought medical attention after realizing he was unable to expel the magnetic beads through urination. The diagnosis was confirmed via CT imaging. Current methods for removing magnetic beads include open cystostomy, laparoscopic intervention, and cystoscopic extraction. According to literature reports, Graziottin et al. (7) successfully retrieved magnetic spheres from a 22-year-old male patient using endoscopic forceps, while Brooks et al. (8) described the extraction of magnetic beads via transurethral cystoscopy with a basket device and a three-pronged grasper in a 26-year-old male. Most patients prefer cystoscopic procedures due to their minimally invasive nature and shorter recovery times.

We purchased similar beads to simulate the surgical procedure and observed that the magnetic force between them was exceptionally strong. Additionally, instruments commonly used in laparoscopy were easily attracted by the magnetic pull. When attempting to remove the magnetic beads using laparoscopic separation forceps through a 22 Fr percutaneous nephroscope sheath, the beads were drawn toward the base of the forceps. This caused the forceps to open excessively, making it difficult to pass them through the sheath. These findings align with previous report findings of difficulties in using stone baskets or foreign body forceps in similar cases (9). Due to the strong magnetic force, the surgeon was unable to remove the magnetic beads using a stone removal basket or foreign body forceps, ultimately requiring a switch to open surgery. We initially attempted to use a loop electrode, commonly used for transurethral resection of the prostate, to separate the beads between the gaps. However, this approach was unsuccessful on the first attempt. Although the loop electrode was not attracted to the beads, it failed to extract them and became significantly deformed in the process. This made it clear that a new tool would be needed for the procedure.

The use of self-made tools for the removal of foreign bodies from the bladder has also been reported. Zeng et al. (10) reported successfully extracting magnetic beads using a self-made sheath with magnetic properties. We reached out to local factories for small magnetic bars, but they were unable to supply them, and the magnetism of the bars diminished after high-temperature sterilization. As a result, we decided to develop a non-magnetic device to extract the beads. We fashioned a tool from orthopedic wire made of non-magnetic stainless steel to prevent magnetic interference (Figure 2). The diameters of the top and bottom of the extractor were approximately 6 and 4 mm, respectively. After sterilization, this device was effectively used to smoothly remove all the magnetic beads.

Previous studies have reported that the treatment approaches adopted for female patients with bladder foreign bodies are similar to those for male patients, both involving the removal of the foreign body through cystoscopy or cystotomy (11). Very few works of literature have reported on the use of self-made tools to treat patients with bladder foreign bodies in females. Moreover, female patients are more likely to insert strip-shaped or rod-shaped foreign bodies into the bladder via the urethra (11, 12). If the foreign body possesses such a configuration, our tool may exhibit reduced efficacy in achieving its retrieve. The simplicity of the tool's design and the easy availability of materials make it particularly suitable for use in primary care hospitals. However, this tool is more appropriate for the removal of spherical foreign bodies than elongated, cord-like objects.

## Conclusions

For additional requirements for specific article types and further information please refer to “article types” on every Frontiers journal page. This study presents a novel method for retrieving magnetic beads from the bladder using a self-made tool. This approach not only reduces the operative duration and complexity but also minimizes surgical trauma. The self-made device employed in this study proved to be relatively simple yet effective in successfully removing the magnetic beads from the bladder.

## Data Availability

The original contributions presented in the study are included in the article/Supplementary Material, further inquiries can be directed to the corresponding author.
